# Effect of thyroid hormone replacement therapy on mortality rate in patients undergoing total or hemithyroidectomy for benign multinodular goitre

**DOI:** 10.1093/bjsopen/zrae012

**Published:** 2024-02-19

**Authors:** Erik Nordenström, Jonas Ranstam, Anders Bergenfelz

**Affiliations:** Department of Clinical Sciences-Lund, Lund University, Sweden; Department of Clinical Sciences-Lund, Lund University, Sweden; Department of Clinical Sciences-Lund, Lund University, Sweden

## Abstract

**Background:**

Thyroid surgery for benign non-toxic nodular goitre is a common endocrine surgical procedure. It is not known whether thyroid hormone replacement therapy following surgery for benign thyroid disease influences mortality or morbidity rates.

**Methods:**

A retrospective observational study was conducted using national registries in Sweden. Overall mortality and morbidity rates were compared for patients with or without thyroid hormone replacement therapy in patients operated on with hemithyroidectomy or total thyroidectomy for a diagnosis of benign non-toxic nodular goitre.

**Results:**

Between 1 July 2006 and 31 December 2017, 5573 patients were included, 1644 (29.5%) patients were operated on with total thyroidectomy and 3929 patients with hemithyroidectomy. In the hemithyroidectomy group, 1369 (34.8%) patients were prescribed thyroid hormone replacement therapy in the follow-up. The patients who underwent hemithyroidectomy and did not use thyroid hormone replacement therapy in the follow-up had a standard mortality ratio of 1.31 (95% confidence interval, 1.09–1.54). The mortality ratio was not increased in patients who underwent total thyroidectomy or hemithyroidectomy and used thyroid hormone replacement therapy. The risk of death analysed by multivariable Cox regression for patients operated on with hemithyroidectomy without later thyroid hormone replacement therapy, adjusted for age and sex, showed an increased hazard ratio of 1.65 (1.19–2.30) compared with hemithyroidectomy with hormone replacement therapy.

**Conclusion:**

Patients subjected to hemithyroidectomy without later hormone replacement therapy had a 30% higher risk of death compared with the normal Swedish population and a 65% increased risk of death compared with patients undergoing hemithyroidectomy with postoperative hormone replacement therapy.

## Teaser

We show in our study a 30% higher risk of death compared with the Swedish population in patients operated on for benign non-toxic nodular goitre with hemithyroidectomy without thyroid hormone replacement therapy after surgery.

Since goitre and thyroid surgery per se are not connected to a higher mortality rate, we think our result in this study is interesting.

## Introduction

Thyroid surgery for benign nodular non-toxic goitre is a common procedure^1^. The incidence of thyroid surgery in Sweden is about 400 operations per million. Depending on the extent of the disease, most commonly either total thyroidectomy (TT) or hemithyroidectomy (HT) is performed^2^, with benign nodular goitre the most common histological diagnosis^2^.

Iatrogenic hyperthyroidism associated with post-surgical treatment of patients operated on for thyroid cancer with thyroxin in suppressive doses has been associated with a higher risk of cardiac arrythmia and the loss of bone mass and fractures^3–6^. Patients with subclinical hyperthyroidism, that is, suppressed levels of thyroid stimulating hormone (TSH) with normal levels of thyroid hormones, also have an increased risk of atrial fibrillation and decreased bone density^7^.

On the other hand, untreated hypothyroidism may lead to serious adverse health effects and death^8–10^. In a meta-analysis, 20% of patients developed hypothyroidism following HT, and in another, it has been described in up to half of the patients subjected to HT^11,12^. Few studies have investigated the association of subclinical hypothyroidism and all-cause death. Some studies have shown a higher risk of cardiovascular events and death associated with hypoparathyroidism in non-surgical patients^8^. Moreover, an association between hypothyroidism and kidney disease, fatty liver disease, diabetes and cancer death has been suggested^13^.

Given previous results, it was hypothesized that there may be an association between thyroid hormone replacement therapy (HRT) and morbidity and mortality rates in patients undergoing thyroid surgery for non-toxic benign nodular goitre. To analyse this, HRT was used as a proxy for hypothyroidism or subclinical hypoparathyroidism.

## Methods

The study was approved by the Swedish Ethical Review Authority (2019-00669).

### Data sources and definitions

The Scandinavian Quality Registry for Thyroid, Parathyroid and Adrenal Surgery (SQRTPA, www.sqrtpa.se) was initiated in 2004 and is recognized by the Swedish National Board for Health and Social Welfare as the National Quality Registry for endocrine surgical procedures in Sweden. Data are entered in an online database. Currently, 36 Swedish units report to the registry, which covers 95% of all thyroid procedures in Sweden^2^. Data quality for the registered patients is evaluated annually via external audit and by comparing registered data to hospital medical records. Audits have demonstrated good data quality^2^.

Data from the SQRTPA was cross-matched with the following central national registries: the Swedish National Registry for Prescribed Drugs, the Swedish National Patient Registry, the Swedish Cancer Registry and the Swedish Cause of Death Register.

#### Swedish National Registry for Prescribed Drugs

The Swedish National Register for Prescribed Drugs was launched 1 July 2005 and contains information about drugs prescribed and dispensed in Sweden. The data from 2005 to 2018 were retrieved. Included variables are age, sex, a unique patient identification number (personal number), Anatomical Therapeutic Chemical (ATC) Classification Code, drug dose, package size, date of prescription, day of dispensing, as well as the prescriber’s profession and practice^14^. The data are entered automatically by direct transfer from computer records at pharmacies.

The defined daily dose (DDD) is a statistical measure of drug consumption, defined by the World Health Organization (WHO) Collaborating Centre for Drug Statistics Methodology. It is defined in combination with the ATC Code drug classification system for grouping related drugs. DDD was used to calculate drug consumption per patient-year based on dispensed prescription after surgery. The prescribed daily dose (PDD) is defined as the average dose prescribed according to a representative sample of prescriptions. HRT was defined as the following ATC codes: H03AA01, H03AA02.

#### The Swedish National Patient Register

As of 1987, the Swedish National Patient Register includes all in-hospital patient care in Sweden. Surgical procedures and diagnoses at all hospitals in Sweden can be retrieved from this registry. Data are entered by direct transfer from computer records in each county. Underreporting of inpatient data has been estimated to be less than 1%^15^.

The ICD-10-SE (International Statistical Classification of Diseases and Related Health Problems – Tenth Revision – Sweden) discharge diagnoses (1987–2018) were retrieved to evaluate prevalent and incident morbidity rates before and after thyroid surgery for groups of patients. Diagnoses were divided into groups: fractures, cardiovascular disease, arrhythmia (all) and atrial fibrillation (*Table S1*).

Further data (1964–2017) on thyroid and parathyroid surgical procedures were retrieved as were discharge ICD codes between 1964 and 2018 for malignant and benign thyroid and parathyroid disease (ICD 10-ICD 7) (*Table S2*).

#### The Swedish Cancer Register

The Swedish Cancer Register is maintained by the National Board of Health and Welfare, and covers the entire population of Sweden. The registry is based on mandatory notification of malignant and certain benign tumours^16^. Patients registered with malignant and benign thyroid and parathyroid tumours between 1958 and 2018 were excluded (*Table S2*).

#### The Swedish Cause of Death Register

The Swedish Cause of Death Register includes those who died during one calendar year and were registered in Sweden at the time of death, regardless of whether the death occurred inside or outside the country. Overall death and disease-specific deaths of the circulatory system (ICD Chapter IX, I00-I99) and neoplasm (ICD Chapter II, C00-D48) were analysed.

##### Inclusion criteria

The study population consisted of patients registered in SQRTPA between 1 July 2006 and 31 December 2017. End of follow-up for the study was 31 December 2018.

Included patients were registered as undergoing first-time thyroid surgery with total TT or HT for non-toxic nodular goitre, and with a final histological diagnosis of benign colloid goitre (defined as the index operation). In the register it is possible to exclude patients that have already been operated on with HT, that is completion HT patients are not included in this study. Sex, age, type of operation, indication for surgery and histology were retrieved from SQRTPA. Data for all these patients were then pulled from all the other described registries.

##### Exclusion criteria

Patients that were treated with thyroid HRT (H03AA01, H03AA02) before the date of the index operation were excluded from analysis. Patients with a malignant or benign thyroid and or parathyroid diagnosis in the cancer registry before or after the date of the index operation were excluded as were surgical thyroid or parathyroid procedures registered in the national patient registry before the index operation.

### Analysis and reporting

Standardized mortality ratios (SMRs) were calculated using 5-year age-, sex- and calendar-year-specific national mortality rates retrieved from the National Swedish Board of Health and Welfare. Time from surgery to death and from surgery to the first post-surgery diagnosis was analysed using the Kaplan–Meier method and the proportional hazards model with the inclusion of adjustments for differences in age and sex. The fulfilment of the assumption of proportional hazards was evaluated using Schoenfeld residuals. No non-proportionality was detected. *P* values lower than 0.05 were considered statistically significant. In the various analyses, patients that moved abroad were not considered to impact on the results. The analyses were performed using Stata release 17.0 (reference: Stata Corp. 2021. Stata Statistical Software: Release 17. Stata Corp LLC, College Station, TX, USA).

## Results

**Fig. 1:**
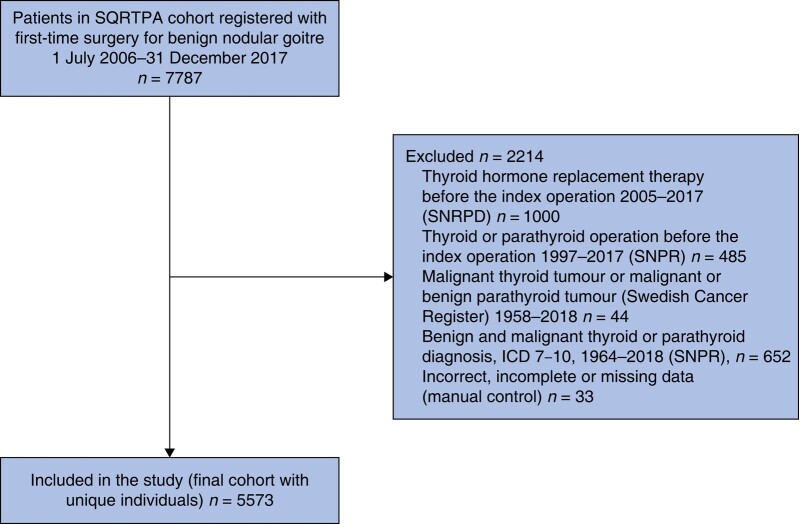
**Study flow chart** SQRTPA, Scandinavian Quality Register for Thyroid, Parathyroid, and adrenal Surgery; SNRPD, Swedish National Register for Prescribed Drugs; SNPR, Swedish National Patient Registry.

During the study interval, 7787 patients underwent first-time surgery for benign nodular goitre. After exclusion criteria were applied, 5573 patients remained in the final cohort (*Fig. 1*). Some 4547 (81.6%) patients were women and the mean(s.d.) age for all patients was 54.6(14.4) years. The median follow-up was 6.1 (1.2–11) years. TT was performed in 1644 (29.5%) and all these patients were prescribed HRT after surgery. Some 3929 patients underwent HT.

Data on the entire group of patients, and the subgroups of patients undergoing TT, and HT with and without HRT in the follow-up therapy is shown in *Table 1*.

**Table 1:** Baseline data in 5573 patients operated on for benign nodular goitre

Variable	Total thyroidectomy	Hemithyroidectomy +HRT	Hemithyroidectomy −HRT
	(*n* = 1644)	(*n* = 1369)	(*n* = 2560)
**Sex**			
Female	1316	1182	2049
Male	328	187	511
Excised thyroid gland weight (g), mean (s.d.)	149 (241)	91 (860)	82 (584)
**Indication for surgery**			
Compression symptoms	1528 (92.9)	1058 (77.3)	946 (76.0)
Excluding malignancy	92 (5.6)	234 (17.1)	413 (16.1)
Suspected malignancy	0 (0)	2 (0.1)	3 (0.1)
Recurrent cyst	12 (0.7)	62 (4.5)	165 (6.4)
Other indication	12 (0.7)	13 (0.9)	33 (1.3)

Values are *n* (%) unless otherwise stated. HRT, thyroid hormone replacement therapy.

**Fig. 2:**
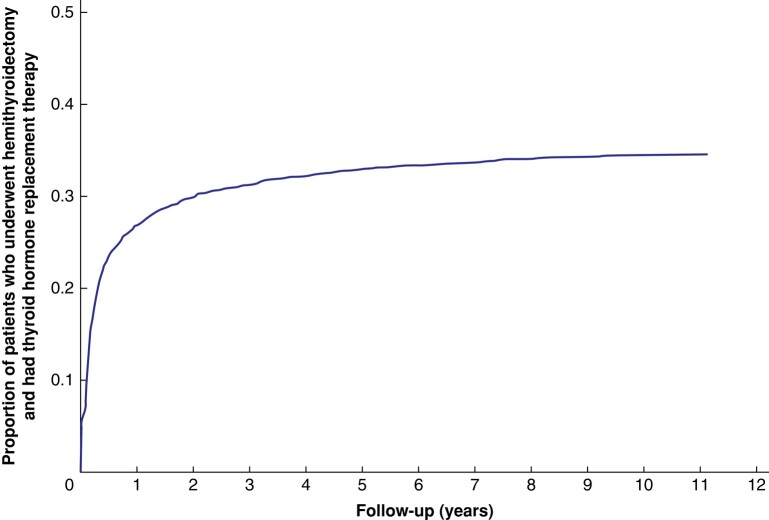
Postoperative prescription of thyroid hormone replacement therapy during the study period in patients operated for benign nodular goitre with hemithyroidectomy

During the study interval, 1369 of 3929 (34.8%) patients subjected to HT were prescribed HRT. The majority of these patients, 712 (52.7%), received HRT within 90 days after surgery and 1169 (86.5%) patients within 1 year after HT. However, prescription of HRT for additional patients continued throughout the study interval (*Table S3* and *Fig. 2*).

Preoperative co-morbidities concerning fractures, cardiovascular disease, arrhythmia and atrial fibrillation are shown in *Table 2*. There were no significant differences between patients treated with or without HRT (*P* > 0.712).

**Table 2:** Preoperative co-morbidity in 5573 patients operated on for benign nodular goitre

Co-morbidity	Total thyroidectomy (*n* = 1664)	Hemithyroidectomy +HRT (*n* = 1369)	Hemithyroidectomy −HRT (*n* = 2560)
Fracture	136 (8)	76 (6)	178 (7)
Cardiovascular disease	92 (6)	59 (4)	150 (6)
Cardiac arrhythmia (all)	108 (6)	71 (5)	149 (6)
Atrial fibrillation	71 (4)	43 (3)	85 (3)

Values are *n* (%). HRT, thyroid hormone replacement therapy.

**Fig. 3:**
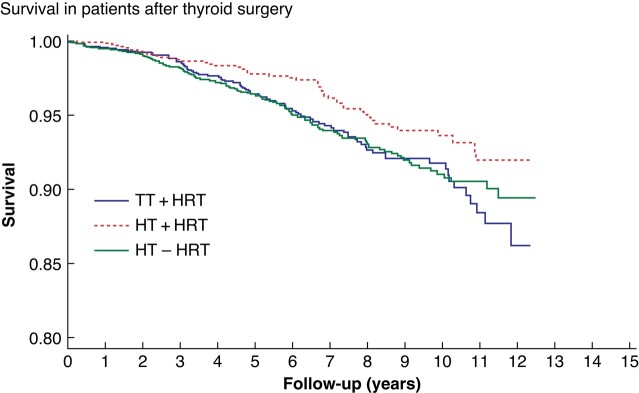
**Kaplan–Meier analysis of survival in patients operated on with hemithyroidectomy (HT) with or without thyroid hormone replacement therapy (HRT) and total thyroidectomy (TT)** Blue line denotes patients operated on with TT + HRT, green line HT − HRT and red line HT + HRT.

During the study interval, 265 (4.8%) patients died. The mortality rate in patients who had undergone TT was 87 of 1644 (5.3%) and for patients who had undergone HT and did not use HRT in the follow-up was 127 of 2560 (5.0%) and for those who used HRT in the follow-up was 51 of 1369 (3.7%) (*Fig. 3*). The SMR for the different patient groups was analysed (*Table 3*). Patients who had undergone HT and did not use HRT therapy in the follow-up had an SMR of 1.31 (1.09–1.54).

**Table 3:** Standardized mortality ratio among patients operated on with total thyroidectomy and hemithyroidectomy with or without thyroid hormone replacement therapy

Group	SMR	Observed	Expected	*P* value	Lower	Upper
TT	1.12	87	77	0.272	0.90	1.37
HT + HRT	0.86	51	59	0.289	0.64	1.12
HT − HRT	1.31	127	97	0.002	1.09	1.54

TT, total thyroidectomy; HT, hemithyroidectomy; HRT, thyroid hormone replacement therapy; SMR, standardized mortality ratio.

In the multivariable Cox regression analysis, adjusted for age and sex, patients operated on with HT without postoperative HRT had an increased risk of death (HR 1.65 (1.19–2.29)) compared with patients with HT with thyroid HRT. In patients who had undergone HT, an increase in the annual DDD for HRT decreased the risk of death (HR 0.71 (0.56–0.91), adjusted for age and sex) compared with patients without postoperative HRT (*Table S4*). A further multivariable Cox regression analysis was performed concerning the impact of the difference between the date of operation and the date of dispensing of HRT adjusted for age and sex. The HR was consistently lower than one and decreased with the number of days, reaching significance after 365 days (*Table 4*).

**Table 4:** Cox regression analysis of the risk of death adjusted for age and sex, and time difference between operation date and the date of dispensing of thyroid hormone replacement therapy

Time difference (days)	HR	Standard error	z	*P* > |z|	95% c.i.	
0–41	0.79	0.17	−1.09	0.275	0.52	1.21
42–90	0.66	0.19	−1.44	0.149	0.37	1.16
91–180	0.59	0.27	−1.15	0.251	0.24	1.45
181–365	0.33	0.24	−1.54	0.123	0.08	1.35
366+	0.27	0.14	−2.60	0.009	0.10	0.72

HR, hazard ratio.

Lastly, the risk of death due to circulatory diseases and neoplasm in patients who had undergone HT and did not use HRT in the follow-up showed an HR of 1.67 (0.89–3.11), and 1.59 (0.96–2.62) respectively (adjusted for age and sex, and with patients operated on with HT and HRT as reference).

After excluding patients with prevalent co-morbidity, defined as selected diagnosis groups (*Table S1*), an analysis of incident co-morbidities after thyroid surgery was performed. There were no significant associations between co-morbidity diagnoses after surgery among the groups studied. Cox regression analysis for selected incident co-morbidity diagnosis, adjusted for age and sex, and with patients with HT and HRT as reference, showed that HR for patients undergoing HT without thyroid HRT was not significantly increased (*Table 5*).

**Table 5:** Cox regression analysis of selected co-morbidity diagnoses and patients operated on with hemithyroidectomy with or without hormone replacement therapy

Type of morbidity	HR	Standard error	z	*P* > |z|	95% c.i.	
Fractures	1.02	0.19	0.12	0.905	0.72	1.46
Cardiovascular disease	1.00	0.21	−0.01	0.990	0.67	1.49
Cardiac arrhythmia	1.08	0.19	0.44	0.657	0.77	1.51
Atrial fibrillation	1.26	0.03	1.18	0.238	0.86	1.86

Analyses were adjusted for age and sex. Reference is hemithyroidectomy with hormone replacement therapy. HR, hazard ratio.

Including patients with TT in the multivariable analysis yielded essentially similar results (data not shown).

## Discussion

Benign non-toxic nodular goitre is a common endocrine disorder^17^. The symptoms are typically related to local compression and surgery is often the only effective treatment. TT or HT are accepted options for surgical treatment and are commonly used worldwide. The thyroid surgical procedure itself is not associated with a higher risk of death, even in older patients^18^, although TT with permanent hypothyroidism has been associated with a higher risk of death and a higher morbidity rate^19,20^. Benign non-toxic goitre has, to the best of our knowledge, not been associated with a higher risk of death.

However, subclinical hypo- or hyperthyroidism in non-surgical patients and in patients treated with TSH suppression after thyroid cancer have been associated with an increased morbidity rate^4,5,9,21–23^.

The study’s results showed patients who underwent HT and did not use HRT in the follow-up had an approximately 30% increased risk of death when compared with the normal Swedish population and 65% increased risk of death when compared with those who used HRT. The risk of death was not increased in patients who underwent TT or in patients operated on with HT who used HRT in the follow-up compared with the normal Swedish population.

In patients that underwent HT, a decreased risk of death with an increase in the annual daily defined doses "DDD" for thyroid hormone was found. Further, the hazard ratio for the risk of death was consistently decreased for the difference in time between operation date and the date of treatment with thyroid hormone, albeit significant only if started more than a year after surgery. This implicates a dose response connection, that is, more HRT lowers the risk of death. The hazard ratios for death in circulatory diseases and neoplasms were not significantly increased. The reason for the increased risk of death in patients operated on with HT and not treated with thyroid HRT cannot as yet be established.

Based on previous studies in non-surgical patients, one plausible explanation is that a group of patients without thyroid HRT were overtly hypothyroid, or suffered from so-called subclinical hypoparathyroidism with increased levels of TSH and normal thyroid hormone levels^21^. A previous study has shown an increase in all-cause death, as well as cardiovascular and cancer death in patients with high normal TSH (1.90–4.50 mIU/l). However, a distinct limitation in the current investigation is that the data set lacks TSH values since this is not registered in the SQRTPA.

There are a number of studies indicating that hypothyroidism may be related to a higher burden of cardiovascular disease but also a higher risk of death^8,11,13,21–23^. Analysis of morbidity rate in the present study was based on selected discharge diagnoses. However, the results showed no association with incident morbidity rate after surgery among the groups of patients. The reason for the increased risk of death in patients with HT without thyroid HRT is therefore unclear and not explained by the current data.

A limitation of the present study is that the diagnoses were collected from the national patient register in Sweden based on discharge diagnoses after in-hospital treatments. It is fair to assume that the true morbidity rate may not have been captured, since many of the diagnoses that were studied are treated in an outpatient setting (for instance cardiac arrhythmia, including atrial fibrillation, and fractures, and even some patients with cardiovascular disease).

It may also be considered if some patients had an increased risk for thyroiditis and autoimmune disease. However, this would probably not have changed the overall results concerning mortality rate. Rather, if there was an imbalance with a higher rate of autoimmune disease in patients undergoing HT with thyroid HRT, this would tentatively entail a higher risk of death in this group of patients.

The strength of the present study is that the cohort was retrieved from a validated quality register for endocrine surgery, and data was cross-matched with national registries, especially the Swedish National Registry for Prescribed Drugs. This registry enables us to capture patients on HRT, when the treatment was prescribed and dispensed at the pharmacy, as well as DDD.

Considering that non-toxic goitre is a benign disease and globally very common, it is alarming that in the group of patients undergoing HT and not treated with thyroid HRT, there is a higher risk of death compared with the normal Swedish population. This risk seems even higher when compared with patients that underwent HT with postoperative thyroid HRT. Until further studies have deduced the mechanism for this increased mortality rate, it is suggested that patients undergoing HT without HRT should be monitored for TSH annually.

## Supplementary Material

zrae012_Supplementary_Data

## Data Availability

Raw data including personal ID numbers were retrieved from different registries described in the Methods section. The data that support the findings of this study are available on request from the corresponding author (E.N.). The data are not publicly available due to restrictions (for example, containing information that could compromise the privacy of research participants). The study protocol can be obtained by contacting the corresponding author. Due to Swedish patient confidentiality laws, the authors will not make individual participant data publicly available. However, a data set with de-identified patient data may be made available at the Swedish National Board of Health and Welfare after application.
