# Supplementary material for: Effect of thyroid hormone replacement therapy on mortality rate in patients undergoing total or hemithyroidectomy for benign multinodular goitre
Source: BJS Open. 2024 Feb 19;8(1):zrae012. doi: 10.1093/bjsopen/zrae012 (PMC10875722; doi:10.1093/bjsopen/zrae012)
Supplement: zrae012_Supplementary_Data [file zrae012_supplementary_data.docx]

Effect of thyroid hormone replacement therapy on mortality rate in patients undergoing total or hemithyroidectomy for benign multinodular goitre

Authors

Erik Nordenström^1^ Jonas Ranstam^1^Anders Bergenfelz^1^

^1^Department of Clinical Sciences Lund University 221 85 Lund Sweden

**Corresponding author:**

Erik Nordenström, [erik.nordenstrom@skane.se](mailto:erik.nordenstrom@skane.se). Department of Clinical Sciences Lund University

**Supplementary Materials - Index**

|  |  |
| --- | --- |
| Supplementary Tables 1-4. | *Pages 2-5* |
|  |  |

Suppl. Table 1. ICD 9 and ICD 10 codes used to investigate morbidity in patients undergoing surgery for benign nodular goitre.

| Morbidity type | ICD 9 | ICD 10 |
| --- | --- | --- |
| Fractures | 800-829,829A,905A-905F, | S02 S12 S22 S32 S42 S52 S62 S72 S82 S92 T02X T08 T10 T12 T142 T902 T911 T912 T921 T922 T931 T932 |
| Cardiovascular disease | 402,410-414,426-428,429 C,429 F,429 G,430-438,440-444,557 | I11, I20-I25, I50, I51.1, I51.2, I51.3, I69-I74, G45-G46, K55, K551, K558, K559 |
| Cardiac arrythmia, including atrial fibrillation | 426-427 | I44-I49, I60-I67 |

Suppl. Table 2. Exclusion of patients with thyroid and parathyroid malignancy and unclear tumours

| Register | ICD10 | ICD 9 | ICD 8 | ICD 7 |
| --- | --- | --- | --- | --- |
| Cancer register | C739  C750 |  |  |  |
| Patient register | C739  C750  D351  D349  D440  D442 | 193  194B  227B  226  237E | 19399  19411  22680  23914  23911 | 194  1951  224 |

Suppl. Table 3. Prescription of thyroid hormone replacement therapy after hemithyroidectomy. Number of new patients on thyroid hormone therapy for each time interval is stated.

| Time after surgery | Number of patients |
| --- | --- |
| 0-41 days | 406 |
| 42-90 days | 306 |
| 91-180 days | 205 |
| 181-365 days | 252 |
| 2 years | 53 |
| 3 years | 38 |
| 4 years | 29 |
| 5 years | 17 |
| 6 years | 12 |
| 7 years | 15 |
| 8 years | 9 |
| 9 years | 6 |
| 10 years | 3 |
| 11 years | 1 |
| Information missing | 17 |
| Total | 1369 |
|  |  |

Suppl. Table 4. Cox regression analysis of mortality and increasing annual daily defined doses of thyroid hormone replacement therapy in patients who underwent hemithyroidectomy for benign nodular goitre

| Cox model | HR | Std. error | z | P>\|z\| | (95 % confidence interval) |
| --- | --- | --- | --- | --- | --- |
| Thyroid Hormone (DDD) | 0.71 | 0.09 | -2.75 | 0.006 | 0.56 0.91 |
| Sex | 1.10 | 0.16 | 0.63 | 0.531 | 0.82 1.46 |
| Age (years) | 1.13 | 0.01 | 19.97 | 0.000 | 1.12 1.15 |

HR= Hazard ratio

DDD= daily defined doses
